# Head and Neck Cancer in Belgium: Quality of Diagnostic Management and Variability Across Belgian Hospitals Between 2009 and 2014

**DOI:** 10.3389/fonc.2019.01006

**Published:** 2019-10-09

**Authors:** Roos Leroy, Cindy De Gendt, Sabine Stordeur, Viki Schillemans, Leen Verleye, Geert Silversmit, Elizabeth Van Eycken, Isabelle Savoye, Vincent Grégoire, Sandra Nuyts, Jan Vermorken

**Affiliations:** ^1^Belgian Health Care Knowledge Centre (KCE), Brussels, Belgium; ^2^Belgian Cancer Registry, Brussels, Belgium; ^3^Centre Léon Bérard, Lyon, France; ^4^Department of Radiotherapy-Oncology, University Hospitals Leuven, University of Leuven, KU Leuven, Leuven, Belgium; ^5^Department of Medical Oncology, Antwerp University Hospital, Edegem, Belgium; ^6^Faculty of Medicine and Health Sciences, University of Antwerp, Antwerp, Belgium

**Keywords:** head and neck cancer, squamous cell carcinoma, quality indicators, quality of care, variability in care, diagnosis, staging

## Abstract

**Aims:** The study assessed the quality of diagnosis and staging offered to patients with a head and neck squamous cell carcinoma (HNSCC) and the variability across Belgian hospitals.

**Methods:** In total, 9,245 patients diagnosed with HNSCC between 2009 and 2014, were identified in the population-based Belgian Cancer Registry (BCR). The BCR data were coupled with other databases providing information on diagnostic and therapeutic procedures reimbursed by the compulsory health insurance, vital status data, and comorbidities. The use of diagnosis and staging procedures was assessed by four quality indicators (QI) (i.e., use of dedicated head and neck imaging studies, use of PET-CT, TNM reporting and interval between diagnosis and start of treatment), for which a target was defined before the analysis. The association between the binary QIs and observed survival was assessed using Cox proportional hazard models adjusted for potential confounders.

**Results:** Overall, 82.5% of patients received staging by MRI and/or CT of the head and neck region before the start of treatment. In 47.6% of stage III–IV patients eligible for treatment with curative intent, a whole-body FDG-PET(/CT) was performed. The proportion of patients whose cTNM and pTNM stage was reported to the BCR was 80.5 and 78.4%, respectively. The median interval from diagnosis to first treatment with curative intent was 32 days (IQR: 19–46). For none of these QIs the pre-set targets were reached and a substantial variability between centers was observed for all quality indicators. No binary QI was significantly associated with observed survival.

**Conclusions:** The four quality indicators related to diagnosis and staging in HNSCC all showed substantial room for improvement. For none of them the pre-set targets were met at the national level and the variability between centers was substantial. Each Belgian hospital received an individual feedback report in order to stimulate reflection and quality improvement processes.

## Introduction

In 2016, there were 2,694 new diagnoses of head and neck cancer in Belgium, 2,005 in males and 689 in females. The mean age at diagnosis was 64 years (1). In Belgium, head and neck cancer is the 4th most frequent tumor in males (6% of all malignancies) and the 11th most frequent in females (2%) (2). Compared to other European countries, Belgium has a very high incidence rate of head and neck cancer: Belgium ranks second for males (after France) and fourth for females (after Denmark, France and the Netherlands) (2). The 5-year relative survival rate for the Belgian 2009–2013 cohort was about 51% in males and 58% in females (2). By 2025, the annual number of patients diagnosed with head and neck cancer is expected to rise to more than 3,000 (3).

In Belgium, adult patients with head and neck cancer can be treated in any acute care hospital, leading to a wide dispersion of care. Only very recently, the first initiative has been taken to concentrate care for adults with complex and/or rare cancers: reference centers have been appointed for pancreatic and esophageal surgery (4, 5).

In recent years, the Belgian Health Care Knowledge Center (KCE) and the Belgian Cancer Registry (BCR) have collaborated intensively in quality improvement initiatives for cancer patients. These start with the development of clinical practice guidelines, followed by the development and the assessment of a set of quality indicators, the formulation of policy recommendations and last but not least individual feedback provided to all hospitals. This improvement cycle has been completed for rectal (in collaboration with PROCARE), breast, testicular, esophageal, gastric, and lung cancer (6–10). Each time clinical experts from Belgian hospitals have been heavily involved.

Given the important burden of head and neck cancer in Belgium and the complexity of its management, this cancer was selected for the following improvement cycle. Evidence-based guidelines for the diagnosis and treatment of squamous cell carcinoma of the oral cavity and the oropharynx, hypopharynx, and larynx were published by KCE in 2014–2015 (11, 12). The present study describes the quality of diagnosis and staging offered in Belgium to patients diagnosed with a squamous cell carcinoma of the head and neck (HNSCC) between 2009 and 2014. The patterns and quality of therapeutic care will be elaborated in a dedicated article.

## Materials and Methods

### Data Collection

Population-based data from the nationwide Belgian Cancer Registry were used. In Belgium, cancer registration is compulsory for hospitals and for pathology laboratories (13). Completeness of incidence has been estimated to be at least 98% of all cancer cases in Belgium from 2004 onwards (14).

The BCR database comprises the following patient and tumor characteristics: age at diagnosis, gender, WHO/ECOG (Eastern Cooperative Oncology Group) performance status score [from score 0 (i.e., fully active, able to carry on all pre-disease performance without restriction) to score 4 (i.e., completely disabled; cannot carry out any self-care; totally confined to bed or chair)], clinical and pathological TNM stages (according to the 6th version of the TNM classification for incidence year 2009 and the 7th version for incidence years 2010–2014) (15, 16), and topography and histology of the tumor (ICD-O-3). The RARECAREnet definition layer 2 of topography and histology combinations was used to classify tumors into the four anatomic groups (i.e., oral cavity, oropharynx, hypopharynx, and larynx, http://www.rarecarenet.eu/). The incidence date was defined as the date of the first histopathological confirmation of the tumor.

The patients' unique social security identification number was used to link the BCR data with (a) data from the Intermutualistic Agency (IMA) providing details on diagnostic and therapeutic procedures reimbursed by the compulsory health insurance starting from 1 January of the year preceding the incidence year, until 31 December of the fifth year after the incidence year; (b) hospital discharge data, comprising (among others) the diagnosis for hospitalization, the principal and secondary diagnoses, available from 1 January of the year preceding the incidence year, until 31 December of the year following the incidence year; and (c) the vital status data of the included patients retrieved from the Crossroad Bank of Social Security (until 14 December 2017). These linkages have been approved by the Sector Committee of Social Security and of Health (Health Section) of the Belgian Privacy Commission (17, 18). At the start of this study, IMA-data were available at the BCR up to June 2016. Based on hospital discharge data, a modified version of the Charlson Comorbidity Index was calculated (19). As only patients with unique HNSCC were included in the study, the categories “Any malignancies, including leukemia, and lymphoma” and “metastatic solid tumor” were left out to calculate the index (20).

Among the 15,339 patients identified in the BCR database with head and neck cancer diagnosed in the period 2009–2014, 12,756 were diagnosed with a squamous cell carcinoma (SCC) of the oral cavity, oropharynx, hypopharynx, or larynx. IMA-data were available for 98.3% of these patients. Patients with multiple invasive tumors (*N* = 3,287) were excluded from the analyses, in order to maximally ensure that recorded diagnostic and therapeutic procedures were indeed performed for HNSCC and not for another malignancy. After additional exclusion of those patients who died around the time of diagnosis or who were lost to follow-up, a final cohort of 9,245 patients with a unique HNSCC was included.

In order to assess the concordance between the diagnostic and therapeutic procedures identified in the administrative database and the information available in the hospitals (e.g., medical files, financial data, considered as “gold standard”), a validation study and subsequent data checks were performed before the analysis of the quality indicators. It led to a further optimization of the code selections to define diagnostic and therapeutic procedures which were used for the calculation of the quality indicators (20).

### Quality Indicators

A long list of potential quality indicators (QIs) was derived from published papers and quality reports, which was supplemented with QIs derived from the KCE guidelines and QIs suggested by the clinical experts. They were scored by a panel of 11 clinical experts, BCR and KCE for their relevance on a 1–5 scale. The in- and exclusion of QIs was further discussed during two consensus meetings. The 33 remaining QIs were then judged for their measurability based on the available data. To that end, the availability of administrative data for every single element of the quality indicator was evaluated. Finally, 12 measurable QIs were retained. Of these, 4 QIs assessed diagnosis and staging, 6 the processes of care and 2 QIs assessed the outcomes of care (post-treatment mortality and survival). Whenever applicable, a target was defined by expert consensus before the analysis of the QI. More information on the selection of the QIs has been published earlier (20).

The present paper focuses on the 4 QIs assessing diagnosis and staging, more precisely on the use of MRI and/or contrast-enhanced CT of the primary site and draining lymph nodes before treatment with curative intent, the use of FDG-PET(/CT) within 6 weeks before the start of treatment, the reporting of TNM staging to the BCR and the time interval between diagnostic confirmation and the start of first treatment with curative intent.

### Hospital Allocation

For the benchmarking of QIs between hospitals, it was essential to identify in which hospital patients received their diagnostic and therapeutic care. In other words, each patient had to be assigned to a center, irrespective of whether the patient had received care in one or in more than one hospital.

In 63% of patients all therapeutic procedures were performed in the same hospital. For the patients who received treatment in more than one hospital, the following hierarchy was given in the assignment of the center of main treatment: center of surgery (with curative intent) if applicable, center of radiotherapy (with curative intent) if applicable, followed by the center of systemic therapy. The center of first treatment took the center of surgery with curative intent, the center of radiotherapy with curative intent and the center of systemic therapy into account. The center where the first of these treatments was performed, was selected as the center of first treatment. In other words, if induction chemotherapy was given in center A and thereafter surgery in center B, the patient was assigned to center A when benchmarking was based on the center of first treatment and was assigned to center B when benchmarking was based on the center of main treatment. The diagnostic acts were not included in the assignment algorithms as it was judged the responsibility of the therapeutic center that all essential diagnostic information was collected before the start of first treatment.

For each QI it was decided before the start of the analysis whether benchmarking between hospitals should be done based on the center of main treatment (QI 1, 2, and 4) or based on the center of first treatment (QI 3) and thus which assignment algorithm had to be applied. More details can be found in an earlier publication (20).

### Statistical Analyses

#### Center Variability

The variability between centers is presented in scatter and funnel plots. In the latter, the estimate of an indicator is plotted on the vertical axis vs. its precision on the horizontal axis. As we were dealing with binary indicators, the estimates were plotted vs. the number of observations of the hospitals, because the precision on the proportion of a binary indicator is proportional to the unit size. The binomial distribution was used for the construction of the 95 and 99% prediction limits; the observed overall indicator result was used as the population or reference value.

As underreporting of TNM stage information (see Results section) may bias the results, those centers which had reported for <50% of their assigned patients stage information to the BCR, were represented differently (i.e., by an open triangle) in the funnel plots.

#### Observed Survival Analysis

Survival time was calculated from the incidence date to the date of death or until the last known date alive. The survival probability over the 0–5 year time interval was modeled with Cox proportional hazards models. Patients surviving beyond 5 years were censored at 5.05 year. Non-proportional hazards between the levels of categorical covariates were evaluated in a univariate way. Detected non-proportional hazards were resolved with a “piece-wise proportional hazards model” (i.e., proportionality assumption holds within time intervals). Then all covariates (i.e., baseline patient case mix variables: gender, age group at diagnosis, WHO performance status, combined stage, anatomic site, the Charlson Comorbidity score, and the number of previous inpatient bed days) were combined in the Cox model, including their non-proportional hazard terms. If the latter were no longer significant, they were dropped in a backwards elimination strategy. Second order interactions between the covariates were evaluated in a backwards elimination model building procedure. The model assumptions were evaluated on the basis of Schoenfeld and generalized Cox-Snell residuals (21, 22); no strong violations were observed. Clustering of patients within hospitals was taken into account by adding hospital as a random effect to the regression model. No imputation techniques were applied in case of missing observations for a covariate; they were assigned to the category “missing.”

The analysis methods were agreed and finalized before the analyses were started. All analyses were performed anonymously and are reported anonymously. Statistical analyses were performed with SAS 9.3 (SAS Institute, Cary, NC, USA).

## Results

### Description of the Cohort at the Time of Diagnosis

Three quarters of the 9,245 included patients were men (Table 1); the mean age at diagnosis was 62.3 years. Sixty percent of the 8,812 patients with available hospital discharge data had no recorded comorbidities. For those with comorbidities, the most prevalent were chronic pulmonary disease (19.4%), diabetes without chronic complications (8.0%), and peripheral vascular disease (5.6%).

**Table 1:** Patient and tumor characteristics at the time of diagnosis.

	**Total (*****N*** **= 9,245)**		**Oral cavity (*****N*** **= 2,665)**		**Oropharynx (*****N*** **= 2,745)**		**Hypopharynx (*****N*** **= 1,137)**		**Larynx (*****N*** **= 2,698)**	
	***N***	**%**	***N***	**%**	***N***	**%**	***N***	**%**	***N***	**%**
**Gender**										
Male	7,017	75.9	1,770	66.4	1,998	72.8	974	85.7	2,275	84.3
Female	2,228	24.1	895	33.6	747	27.2	163	14.3	423	14.3
**Age group**										
<50 years	930	10.1	339	12.7	319	11.6	84	7.4	188	7.0
50–59 years	3,058	33.1	869	32.6	1,013	36.9	437	38.4	739	27.4
60–69 years	3,047	33.0	772	29.0	916	33.4	411	36.2	948	35.1
70–79 years	1,481	16.0	410	15.4	364	13.3	146	12.8	561	20.8
≥80 years	729	7.9	275	10.3	133	4.9	59	5.2	262	9.7
**Adapted charlson comorbidity index**										
0^*^	5,359	60.8	1,548	61.8	1,598	61.6	609	55.4	1,604	61.3
1–2^*^	2,747	31.2	777	31.0	769	29.7	393	35.8	808	30.9
3–4^*^	557	6.3	145	5.8	183	7.1	69	6.3	160	6.1
>4^*^	149	1.7	35	1.4	43	1.7	28	2.5	43	1.6
No data available	433		160		152		38		83	

* *The % for the adapted CCI were calculated excluding the missing data*.

**Figure 1:**
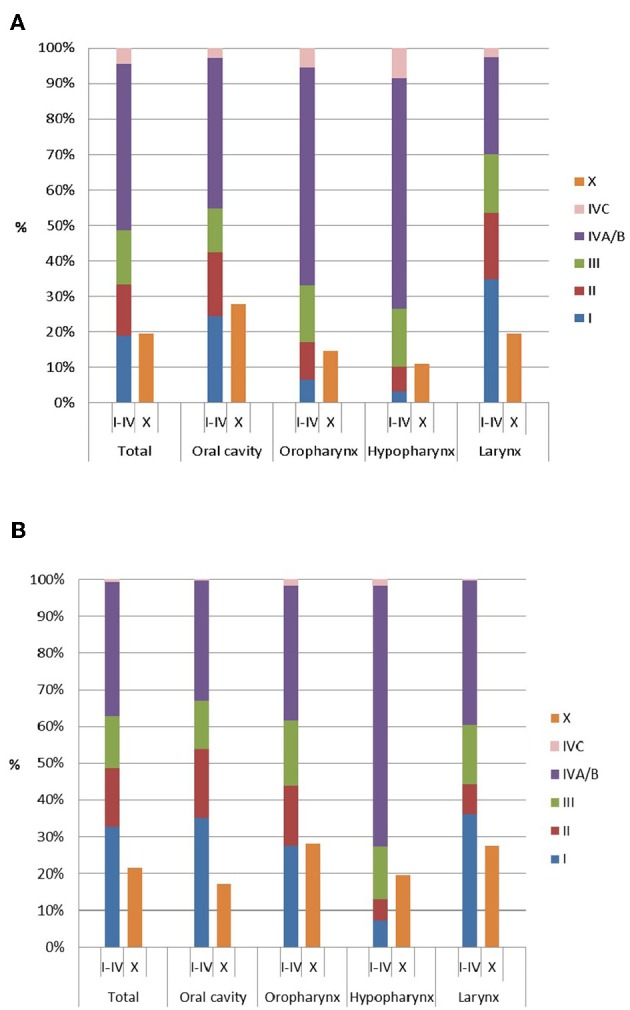
Distribution of **(A)** clinical and **(B)** pathological stage (of surgically treated patients) by anatomic site.

Two thirds of the patients in whom clinical stages were reported were diagnosed in an advanced stage of the disease (cIII–IV, 66.7%; Figure 1), but this proportion varied considerably among the different anatomic sites. For all HNSCC patients who had surgery and for whom the pathological stage was reported to the BCR, pathological stage I and IVA were most common (32.8 and 35.6%, respectively). Yet, for hypopharyngeal SCC the majority of patients (68.5%) were diagnosed with a p-stage IVA.

The 9,175 HNSCC patients who could be assigned to a center of main treatment, were treated in 99 different centers.

### Main Diagnostic and Staging Procedures

The most frequent imaging exams performed in the time span 3 months before until 3 months after the incidence date, were CT of the neck (92.5%) and RX of the thorax (73.3%; see Table 2). A MRI of the neck was performed in 30.1% of cases, ranging from 19.3% in laryngeal SCC to 37.7% in oropharyngeal SCC patients. PET(/CT) was performed in 47.9% of the total study population, with an obvious difference between the different anatomic sites (36.0% in laryngeal SCC vs. 62.3% in hypopharyngeal SCC). The most commonly performed endoscopic procedure was tracheoscopy/laryngoscopy (84.9%), which was performed in 60.0% of patients with oral cavity SCC and in 98.6% of patients with laryngeal SCC. For almost all patients (98.7%), a biopsy of the primary tumor was taken. A multidisciplinary team (MDT) meeting was recorded for 82.3% of the total study population. Additional analyses in the BCR database (results not presented) revealed that over the time span 2004–2014, the proportion of HNSCC patients discussed during a MDT meeting increased substantially. The most pronounced advances were recorded for laryngeal and oropharyngeal SCC: from 42 and 46% in 2004 to 84 and 83% in 2014, respectively.

**Table 2:** Diagnostic and staging procedures performed within 3 months around the incidence date of HNSCC.

**Category**	**Total (*****N*** **= 9,245)**		**Oral cavity (*****N*** **= 2,665)**		**Oropharynx (*****N*** **= 2,745)**		**Hypopharynx (*****N*** **= 1,137)**		**Larynx (*****N*** **= 2,698)**	
	***N***	**%**	***N***	**%**	***N***	**%**	***N***	**%**	***N***	**%**
**Imaging**										
RX thorax	6,772	73.3	2,086	78.3	1,921	70.0	892	78.5	1,873	69.4
RX swallow mechanism /esophagus	682	7.4	45	1.7	162	5.9	171	15.0	304	11.3
RX larynx	108	1.2	12	0.5	15	0.6	31	2.7	50	1.9
CT neck	8,548	92.5	2,289	85.9	2,644	96.3	1,111	97.7	2,504	92.8
CT skull	1,700	18.4	494	18.5	554	20.2	272	23.9	380	14.1
MRI neck	2,783	30.1	920	34.5	1,035	37.7	307	27.0	521	19.3
MRI head	589	6.4	274	10.3	188	6.9	48	4.2	79	2.9
PET(/CT)	4,425	47.9	1,093	41.0	1,653	60.2	708	62.3	971	36.0
Ultrasound neck	1,763	19.1	428	16.1	726	26.5	304	26.7	305	11.3
Ultrasound abdomen	3,178	34.4	991	37.2	1,005	36.6	426	37.5	756	28.0
**Endoscopy**										
Tracheoscopy/laryngoscopy	7,844	84.9	1,598	60.0	2,478	90.3	1,108	97.5	2,660	98.6
Bronchoscopy	1,874	20.3	465	17.5	582	21.2	312	27.4	515	19.1
Nasal endoscopy	745	8.1	147	5.5	275	10.0	121	10.6	202	7.5
**Screening digestive tract**	5,445	58.9	1,345	50.5	1,786	65.1	885	77.8	1,429	53.0
**Histopathology**										
Biopsy of primary tumor	9,127	98.7	2,640	99.1	2,697	98.3	1,110	97.6	2,680	99.3
Lymph node biopsy	320	3.5	68	2.6	156	5.7	46	4.1	50	1.9
Cytology	1,746	18.9	354	13.3	711	25.9	303	26.7	378	14.0
**Multidisciplinary team meeting**	7,608	82.3	2,071	77.7	2,358	85.9	1,009	88.7	2,170	80.4

*HNSCC, head and neck squamous cell carcinoma*.

### Quality Indicator 1—Proportion of Non-metastatic HNSCC Patients Who Underwent MRI and/or Contrast-Enhanced CT of the Primary Site and Draining Lymph Nodes Before Treatment With Curative Intent

According to the guidelines, MRI is the preferred technique for primary T- and N-staging in oral cavity SCC and highly recommended in hypopharyngeal, laryngeal, and oropharyngeal SCC. However, a contrast-enhanced CT can replace MRI when (a high quality) MRI is technically impossible, likely to be distorted, or not timely available (11, 12). Overall, 25.4% of patients were staged by MRI and another 57.1% by CT, within 6 weeks before the start of treatment. The overall result (i.e., 82.5%) was below the target set at 90% (Table 3).

**Table 3:** Overview of 4 quality indicators for diagnosis and staging of HNSCC patients diagnosed in 2009–2014.

**Number**	**Quality indicator**	**n/N**	**Result (%)**	**Target (%)**
QI 1	Proportion of non-metastatic HNSCC patients who underwent MRI and/or contrast-enhanced CT of the primary site and draining lymph nodes before treatment with curative intent	6,630/8,039^*^	82.5	90
QI 2	Proportion of HNSCC patients who underwent FDG-PET(/CT) within 6 weeks before start of treatment Stage I–II	544/2,372^**^	22.9	≤5
	Stage III–IV	2,198/4,619^**^	47.6	≥90
QI 3	A. Proportion of HNSCC patients whose cTNM stage was reported	7,444/9,245	80.5	95
	B. Proportion of HNSCC patients who had surgery, whose pTNM stage was reported	2,758/3,518	78.4	95
QI 4	A. Median time between incidence date and start of first treatment with curative intent	(*N* = 8,040^***^)	32 days (IQR: 19–46)	ND

ND, not defined;

* 328 patients with distant metastases and 878 patients who did not receive treatment with curative intent within six months of the incidence date were excluded from the analyses;

** 1801 patients with missing cTNM information were excluded from the analyses;

*** *327 patients with distant metastases and 878 patients who did not receive treatment with curative intent within six months of the incidence date were excluded from the analyses*.

**Figure 2:**
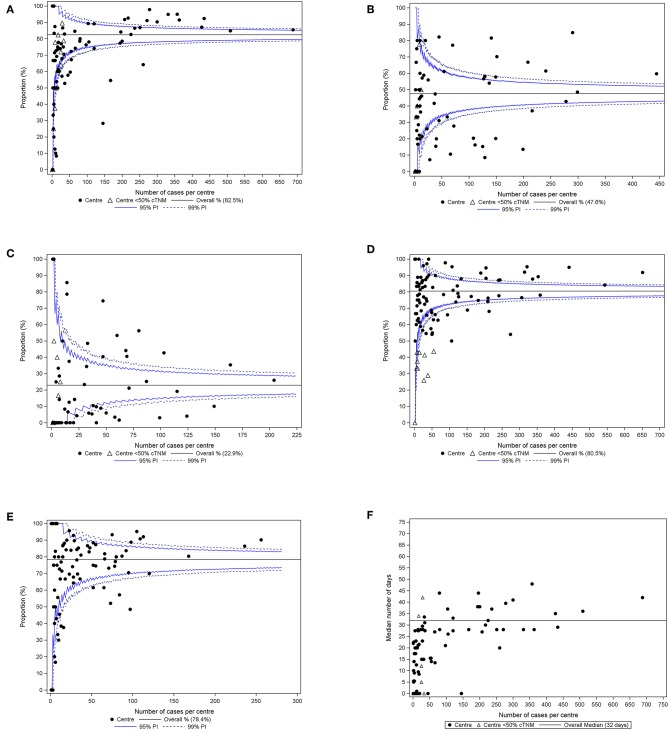
**(A)** Proportion of HNSCC patients who received treatment with curative intent in whom a MRI and/or CT was obtained within 6 weeks before the start of the first treatment, by center of main treatment. Ninety-six centers reported in the funnel plot; centers which reported for <50% of their assigned patients cTNM to the BCR, are represented by an open triangle. **(B)** Proportion of clinical stage III-IV HNSCC patients who underwent treatment with curative intent in whom a whole-body FDG-PET(/CT) was obtained within 6 weeks before start of the first treatment, by center of main treatment. Eighty-seven centers reported in the funnel plot; centers which reported for <50% of their assigned patients cTNM to the BCR, are represented by an open triangle. **(C)** Proportion of clinical stage I–II HNSCC patients in whom a whole-body FDG-PET(/CT) was obtained within 6 weeks before start of the first treatment, by center of main treatment. Eighty-six centers reported in the funnel plot; one patient is not included in the analyses as he/she could not be assigned to the center of main treatment, but his/her data are included in the analyses for the overall result; centers which reported for <50% of their assigned patients cTNM to the BCR, are represented by an open triangle. **(D)** Proportion of HNSCC patients whose cTNM was reported to the BCR, by center of first treatment. One hundred and one centers reported in the funnel plot; 132 patients were not included in the analyses because they could not be assigned to a center of first treatment, but their data are included in the analyses for the overall result; centers which reported for <50% of their assigned patients cTNM to the BCR, are represented by an open triangle. **(E)** Proportion of HNSCC patients whose pTNM was reported to the BCR, by center of main treatment. Ninety-six centers reported in the funnel plot. **(F)** Time from incidence date to first treatment with curative intent, by center of main treatment. Ninety-six centers reported in the scatter plot; centers which reported for <50% of their assigned patients cTNM, are represented by an open triangle. PI, prediction interval.

About 10 centers fell below the 99% prediction interval; only 15 centers (16%) reached the target (Figure 2A).

### Quality Indicator 2—Proportion of HNSCC Patients Who Underwent FDG-PET(/CT) Within 6 Weeks Before Start of Treatment

A whole-body FDG-PET(/CT) is recommended for the evaluation of metastatic spread at distant sites and/or the detection of second primary tumors in patients with stage III–IV HNSCC while it is not recommended in stage I–II HNSCC (11, 12). In less than half of stage III–IV patients who underwent treatment with curative intent (47.6%), a whole-body FDG-PET(/CT) was performed, which was far below the target (≥90%, Table 3). On the other hand, 22.9% of stage I–II patients who underwent any treatment had a FDG-PET(/CT), which is largely above the target (≤ 5%) and deemed thus unnecessary.

For FDG-PET(/CT) in stage III–IV patients, no center reached the target (Figure 2B), while 42 out of 86 centers performed a whole-body FDG-PET(/CT) in more than 5% of the assigned stage I–II patients (Figure 2C).

### Quality Indicator 3—Proportion of HNSCC Patients Whose TNM Stage Information Was Reported to the Belgian Cancer Registry (BCR)

As this study is based on administrative data (hence no access to medical files), a proxy approach was used to assess the staging of the included patients: the completeness of the data transferred to the BCR was evaluated. For 80.5% of patients with HNSCC the cTNM stage was reported to the BCR, which was below the target defined by the clinical experts (95%, Table 3). Overall, the pTNM stage of 78.4% of patients who underwent surgery with curative intent was reported. For cTNM as well as pTNM, the proportion of patients whose staging information was reported to the BCR was much higher among those who were discussed during a MDT meeting (cTNM: 87.3 vs. 49.0%; pTNM: 81.7 vs. 64.5%).

About 15% of the centers were situated below the 99% prediction interval for clinical staging (Figure 2D) and about 11% for pathological staging (Figure 2E). Only a limited number of centers reached the target of 95%.

### Quality Indicator 4—Median Time Between Incidence Date and Start of First Treatment With Curative Intent

Overall, the median interval from diagnosis to first treatment with curative intent was 32 days [Interquartile range (IQR): 19–46; Table 3]. When surgery was the main treatment modality this lag time was shorter (24 days, IQR: 1–40); the median delay to start of primary radiotherapy was 36 days (IQR: 26–49). Patients who received their first treatment in the same center where the diagnosis was confirmed, were treated within a shorter time frame (26 days, IQR: 10–39) than patients who were referred to another center for treatment (37 days, IQR: 26–52).

A large variability was observed between centers; the median time from incidence to treatment varied between 0 and 50 days when benchmarking was done based on the center of main treatment (Figure 2F).

### Association Between Quality Indicators and Observed Survival

In final analyses, the association between the binary quality indicators and observed survival was assessed, taking the baseline patient case mix variables into account. As is presented in Table 4, none of the associations was statistically significant.

**Table 4:** Association between quality indicators and observed survival.

**Quality indicator**	Hazard ratio^*^ [95% CI]
Proportion of non-metastatic HNSCC patients who underwent MRI and/or contrast-enhanced CT of the primary site and draining lymph nodes before treatment with curative intent	1.10 [0.99, 1.22]
Proportion of HNSCC patients who underwent FDG-PET[/CT] within 6 weeks before start of treatment—Stage III–IV	1.00 [0.92, 1.09]
Proportion of HNSCC patients whose cTNM stage was reported	1.12 [0.99, 1.27]
Proportion of HNSCC patients who had surgery, whose pTNM stage was reported	0.86 [0.73, 1.01]

* *Hazard Ratios for all-cause death (yes vs. no) were corrected for baseline patient case mix variables: gender, age group at diagnosis, WHO performance status, combined stage, anatomic site, the Charlson Comorbidity score and the number of previous inpatient bed days*.

## Discussion

Three quarters of this national cohort of patients with HNSCC were male and nearly 60% of the study population was 60 years or older at the time of diagnosis. These observations are in line with other publications that illustrate that head and neck cancers occur predominantly in males and the older segment of the population (23, 24). Also in this study population the majority of patients was diagnosed late, which is a major concern in head and neck cancers where early detection is difficult to achieve (23, 25). One of the factors that contributes to the late diagnosis of head and neck cancers is patient delay (26, 27).

The complexity of head and neck cancers, the close proximity of functionally important anatomic structures and the fact that patients are often elderly with medical comorbidities, necessitate the coordinated professional efforts of a highly specialized multidisciplinary team to guarantee the best oncological outcome and to prevent and adequately manage any adverse effect of treatment (24, 28). Evidence from recent years illustrates that this multidisciplinary approach is beneficial for head and neck cancer patients and leads to improved survival rates (29–33). In this study group more than 80% of patients were discussed during a MDT meeting. Probably, the real frequency of MDT meetings is underestimated, due to (among others) the reimbursement rules (34). For instance, from 2003 to 2010 only one MDT meeting per patient per calendar year was reimbursed by the health insurance and thus “traceable” in the administrative data (34). Yet, one should realize that these data do not reveal whether the MDT meeting was attended by sufficiently experienced medical and paramedical experts and whether it also resulted in a multidisciplinary approach throughout the whole care process (20).

Precise specification of clinical and pathological stage is an essential step in the clinical cancer pathway as it helps in planning the treatment or the renouncement of treatment (so that under- or overtreatment can be avoided), but it aids as much in predicting the patient's prognosis (35, 36). Still, the four process indicators related to diagnosis and staging which were assessed in the present study all showed substantial room for improvement. Overall 82.5% of non-metastatic patients who received treatment with curative intent were staged with MRI and/or CT of the head and neck area before the start of the first treatment, which was below the pre-set target of 90%. Yet, the results are in the order of what was observed in England and Wales (2013–2014) (37), or in Ontario (2010) (38), where 17.8 and 28%, respectively, of all diagnosed patients did not obtain staging information with CT, MRI, PET(/CT), or ultrasound prior to treatment. Although MRI is preferred for the staging of oral cavity SCC and highly recommended in the other anatomic sites, CT was twice as frequently performed as MRI in Belgium (57.1 vs. 25.4%, respectively). This may in part be explained by differences in availability of both technologies: the number of registered CT scans is currently at least twice the number of MRI scans. Obligatory registration of this equipment only started in 2016 (39), but one can assume that a similar ratio was also relevant for the period 2009–2014. In addition, the medical team may opt for a CT as the longer duration of a MRI examination may cause difficulty with breathing and may often be associated with movement artifacts. But also, performing a MRI of the larynx and hypopharynx requires an experienced radiologist coupled with adapted high end hard (MR and coils) and software (right sequences and software to speed-up examination) (40).

Even though a whole-body FDG-PET(/CT) is recommended in patients with clinical stage III–IV HNSCC (11, 12), it was performed in less than half of this subgroup within 6 weeks before start of the first treatment with curative intent. Several factors may explain these sub-optimal results. First of all, until 2016, staging of primary head and neck cancer was not included in the list of reimbursed indications for FDG-PET(/CT) and during the study period the overall availability of and access to FDG-PET(/CT) in Belgium was limited. In addition, there may be a slight underestimation of the real number of patients who underwent FDG-PET(/CT), as in some patients this examination may have been performed in the referring center and may have fallen outside the time frame of 6 weeks set for this quality indicator. Last, some patients may have undergone FDG-PET(/CT) in the frame of a clinical study (e.g., imaging study), which is then not included in the administrative database used for the present study as it could not be billed. Yet, no less than 22.9% of patients with early stage HNSCC, for whom this exam is not recommended, had a whole-body FDG-PET(/CT). The results illustrate that more efforts are needed in this field so that the right group of patients benefits from this diagnostic tool but equally that unnecessary exposure to irradiation and unnecessary use of costly equipment can be avoided.

In Belgium, hospitals are legally bound to report all new cancer diagnoses to the BCR, whether or not the patient is discussed during a MDT meeting (41). In parallel, the law stipulates that pathology laboratories have to transfer (among others) stage information of the pathology specimens they have received to the BCR (13). It is thus difficult to understand that clinical and pathological stage information was not reported for 19.5 and 21.6% of patients, respectively. Part of the lower than expected reporting on cTNM may be found in the underreporting of Tis and T1, especially in case of laser resections and excisional biopsies of the oral cavity. But also, in those cases where no malignancy was suspected before the surgical intervention cTNM may not have been reported to the BCR. Difficulties in accurate staging was also illustrated in other countries (37).

Timely treatment of (head and neck) cancer is essential, not only to increase the chance for cure and to increase survival rates, but also to alleviate the symptoms as soon as possible (42, 43). Half of the study population received the first treatment with curative intent within 32 days. Although the results compared favorably with those reported in other European countries (37, 44, 45), inspiration for a further improvement in this field can for instance be obtained in Denmark, where organizational reforms coupled with the implementation of a fast track program resulted in significant reductions of waiting times between diagnosis and treatment, for both surgery and radiotherapy (43). The observation that the time delay for radiotherapy was longer than for surgery, may be explained by the fact that for radiotherapy the preparatory phase needs more time. In addition, patients who will receive radiotherapy in the head and neck region, should have a thorough pre-radiotherapy dental assessment and, when indicated, treatment (46, 47). In case tooth extractions are performed, it is important to allow sufficient healing time prior to the commencement of radiotherapy. Patients who received their first treatment in the same center where the diagnosis was confirmed, started their treatment within a shorter time frame than their peers who were referred. These data should not be misinterpreted to suggest that referring patients is detrimental. The improved survival at academic and comprehensive centers is indicative of the opposite (48). It has been suggested that treatment of head and neck cancers in high volume centers mitigates some portion of mortality risk due to prolonged time to treatment, but referral of patients should be well-organized to avoid harmful delays (48).

The funnel and scatter plots indicate that for the four QIs under study the variability between centers was substantial. For all indicators the variability between centers was more than what could be expected based on random variability. For one indicator (FDG-PET(/CT)) in advanced stage disease (Figure 2B), none of the centers achieved the set target. In order to improve the current situation, each Belgian hospital received an individual feedback report with its own results for the QIs, benchmarked to those of all other hospitals (which were kept blinded). The concept is that mirror-information may act as a catalyst for quality improvement in care, which ultimately may lead to a better quality of care offered to patients with head and neck cancer. In addition, it can be speculated that the centralization of care for head and neck cancer in a limited number of hospitals (at present adult patients with head and neck cancer can be treated in any acute care hospital in Belgium), will further reduce the variability between centers. At least in a Canadian study, adherence rates to guideline-recommended processes of care in the surgical management of patients with head and neck cancer were higher in high (surgeon and hospital surgical) volume centers than in low volume centers (38).

The observation that none of the binary diagnosis and staging related QIs was significantly associated with all-cause observed survival, after correction for baseline case-mix variables, is not surprising. Many other process (e.g., type of treatment, timing of treatment) and structure (e.g., hospital volume, equipment, financing) indicators may have a more pronounced impact on survival in head and neck cancer. They will be the subject of further analyses.

One of the major strengths of this study is that the quality of diagnosis and staging for 9,245 patients diagnosed with a single squamous cell carcinoma of the oral cavity, oropharynx, hypopharynx, or larynx could be assessed in a population-based database, covering more than 98% of all cancer cases in Belgium (14). Yet, the major strength of the study is at the same time also its major weakness. The interpretation of the administrative data was not always straightforward, due to among others the lack of specificity of the claims data (e.g., vague codes which may refer to a diagnostic as well as a therapeutic procedure), but also due to the careless registration in some hospitals (e.g., cTNM, pTNM, start date of radiotherapy).

In conclusion, the four process indicators related to diagnosis and staging in head and neck squamous cell carcinoma all showed substantial room for improvement. For none of them the pre-set targets were met at the national level and the variability between centers was substantial. Individual feedback reports have been sent to each Belgian hospital in order to stimulate reflection and quality improvement processes.

## Data Availability Statement

The datasets generated for this study will not be made publicly available. The datasets for this manuscript are not publicly available because it concerns coded data at an individual level, i.e., data from the Belgian Cancer Registry (BCR) enriched with health claims data obtained via the Intermutualistic Agency (IMA/AIM). Authorization from the Belgian Data Protection Authority is necessary to access the data. Requests to access the datasets should be directed to Dr. Liesbet Van Eycken, director Begian Cancer Registry, elizabeth.vaneycken@kankerregister.org.

## Ethics Statement

Ethical review and approval was not required for the study on human participants in accordance with the local legislation and institutional requirements. Written informed consent for participation was not required for this study in accordance with the national legislation and the institutional requirements.

## Author Contributions

RL, CD, SS, VS, LV, GS, EV, IS, VG, SN, and JV contributed to the conception and design of the study and performed data interpretation. CD, VS, and GS analyzed the data. RL wrote the first draft of the manuscript. All authors contributed to manuscript revision, read and approved the submitted version.

### Conflict of Interest

The authors declare that the research was conducted in the absence of any commercial or financial relationships that could be construed as a potential conflict of interest.
